# Decision‐making in dementia care: insights from Brazilian family caregivers

**DOI:** 10.1002/alz70858_102946

**Published:** 2025-12-26

**Authors:** Barbara Pires de Andrade Lage Cabral, Maria Rita de Lima Silva, Maria Clara Pereira da Silva, Thais Helena Machado, Marcella Guimarães Assis

**Affiliations:** ^1^ Universidade Federal de Minas Gerais, Belo Horizonte, Minas Gerais, Brazil

## Abstract

**Background:**

The aging population in Brazil reached 10.49% in 2022, with projections indicating growth to 25.49% by 2060. This demographic shift is associated with an increase in chronic diseases, including dementia—a progressive neurodegenerative condition. Family caregivers play a critical role in decision‐making, which becomes more complex as dementia progresses. This study aimed to explore the perspectives of Brazilian family caregivers on decision‐making in dementia care.

**Method:**

This study is part of qualitative, descriptive, and exploratory research on family caregivers’ experiences with Advance Care Planning (ACP), which included 63 family caregivers of older adults with dementia in Belo Horizonte, Brazil. Participants were recruited through outpatient clinics and snowball sampling. Semi‐structured interviews addressed decision‐making processes and sociodemographic data. Data analysis followed reflexive thematic analysis.

**Result:**

Family caregivers demonstrated diverse approaches to decision‐making. Some prioritized the autonomy of their loved ones, even when cognitive decline posed challenges: “[…] I respect [her wishes]. She decides how she wants to be treated and doesn’t accept being forced into anything. She questions, analyzes […] She goes to the doctor she likes because she wants to be treated well” (I13, nephew, 52y). Others took full control, prioritizing what they considered best for the person with dementia: “If I had to decide today, I wouldn’t consider [my mother's wishes]. I would stick with my own decision. Today, I feel like I’m the parent […]” (I60, daughter, 51y). A third group adopted a balanced approach, negotiating and adapting decisions to align caregiving needs with their relative's preferences: “I would have to adapt to her. She's ill, and I need to adapt. For example, she sometimes chooses who will bathe her […]” (I07, sister, 74 y). These findings highlight the complex interplay of caregiver autonomy, relational dynamics, and practical caregiving demands.

**Conclusion:**

Decision‐making in dementia care is multifaceted and influenced by the progression of the disease, caregiver stress, and access to information. ACP can help caregivers navigate this process, reducing conflicts and ensuring dignity for individuals with dementia.

